# Circular RNA hsa_circ_0005519 contributes to acute kidney injury via sponging microRNA-98-5p

**DOI:** 10.1186/s12882-024-03544-8

**Published:** 2024-03-19

**Authors:** Linsen Jiang, Manxin Huang, Jun Ge, Xuefeng Zhang, Ye Liu, Hang Liu, Xiaoming Liu, Lili Jiang

**Affiliations:** 1 Department of Nephrology, The Second Affiliated Hospital of Soochow University, Suzhou, 215000 China; 2 School of Medicine, Jianghan University, Wuhan, 430056 China; 3 Department of Nephrology, Yantai Affiliated Hospital of Binzhou Medical University, No. 717, Jinbu Street, Muping District, Yantai, 264100 China; 4 Department of Pharmacy, Yantai Affiliated Hospital of Binzhou Medical University, Yantai, 264100 China; 5 Department of Nephrology, Youyang Tujia and Miao Autonomous County People’s Hospital, No.102, Middle Road, Taohuayuan Avenue, Taohuayuan Street, Youyang County, Chongqing, 409800 China

**Keywords:** CircRNA, MiRNA, Acute kidney injury, Cell viability, Inflammation

## Abstract

**Background:**

This study intends to explore the role and molecular mechanism of hsa_circ_0005519 in acute kidney injury (AKI).

**Methods:**

We conducted reverse transcription-qPCR for human serum to determine levels of hsa_circ_0005519 in AKI patients and healthy controls. Hsa_circ_0005519 was inhibited for expression in HK-2 cells using specific siRNAs. A number of techniques, MTT and ELISA assays, were used to analyze the potential role of hsa_circ_0005519 in cell viability, oxidative stress, and inflammation of LPS-induced HK-2 cells.

**results:**

The serum of patients with AKI exhibited a significant increase in hsa_circ_0005519 expression, compared with healthy controls. Hsa_circ_0005519 was knockdown by siRNA, and its knockdown led to cell viability increase in LPS-induced HK-2 cells. Inhibition of hsa_circ_0005519 can reverse the TNF-α, IL-6 and IL-1β increase in LPS-induced HK-2 cells. Inhibiting hsa_circ_0005519 led to downregulation of MPO and MDA levels. MiR-98-5p was a downstream miRNA for hsa_circ_0005519. MiR-98-5p can offset the effects of hsa_circ_0005519 on LPS-induced HK-2 cells. IFG1R was a target gene for miR-98-5p.

**Conclusions:**

These findings indicate that the highly expressed hsa_circ_0005519 plays a promoting role in AKI.

**Supplementary Information:**

The online version contains supplementary material available at 10.1186/s12882-024-03544-8.

## Background

Acute kidney injury (AKI) refers to an abrupt, often silent, deterioration in renal function and kidney structure, manifested as a rise in serum creatinine and/or fall in urine output [1]. It is the clinical manifestation of several disorders, such as hypovolaemia, sepsis, obstructive uropathy, nephrotoxins, or a variety of parenchymal renal diseases, that affect the kidney acutely [2]. An increasing incidence of AKI occurs globally, in both high-income and low-income countries. This increase is associated with longer periods of hospital stay, as well as high costs [3]. AKI is responsible for about 1.7 million deaths per year worldwide, becoming a major global public health problem [3, 4]. However, till now, few preventive options or specific therapies have emerged for AKI treatment or expedite recovery. Apart from supportive treatment, patients usually receive renal replacement therapy if acute kidney injury is severe [5]. New diagnostic techniques might help with AKI early diagnosis. Novel biomarkers are urgently needed for early AKI diagnosis and the prediction of acute kidney injury recovery.

The cellular response to injury is heterogeneous, involving apoptosis or viability [6]. Recently, an increasing body of evidence suggests that renal tubular cells are able to maintain viability, which may be related to some identified genes or epigenetic regulation [7]. Studies have demonstrated a role for epigenetic regulation, involving remarkable changes in the expression of various non-coding RNAs (ncRNAs), in the process of AKI and kidney repair [8]. Circular RNAs (circRNAs), a newly described type of ncRNA, have been studied increasingly in AKI [9]. A series of specific circRNAs have been implicated in the pathogenesis of AKI [10]. For instance, Circ-Snrk is involved in AKI development may through MAPK signaling pathway [11]. Emerging research suggests a potential for circRNAs as novel diagnostic or outcome-predicting biomarkers of AKI. ciRs-126 may act as a predictor of mortality in patients with AKI [12]. Urinary hsa_circ_0001334 might serve as a novel non-invasive marker in diagnosing acute rejection [13]. Further investigation into circRNAs in AKI will not only provide novel insights into the mechanisms of AKI, but also might lead to new strategies for the identification and treatment of this disease.

Hsa_circ_0005519 (hsa_circSNX13_023), with sorting nexin-13 (SNX13) as its host gene, has been identified involving in inflammation during asthma [14]. Hsa_circ_0005519 has been screened as an upregulated circRNA in neonatal AKI [15]. However, its detailed role in AKI and the potential mechanism haven’t been clarified. Here, we intend to explore the clinical significance of hsa_circ_0005519 in AKI, and whether hsa_circ_0005519 influences the AKI progress. The potential molecular mechanisms involved in the hsa_circ_0005519 function in AKI were preliminarily elucidated.

## Methods

### Subjects and samples

This retrospective study was performed using existing clinical information and samples from eligible subjects in Yantai Affiliated Hospital of Binzhou Medical University from December 2021 to December 2022. This study selected trauma-induced kidney injury patients from the cohort who had at least one outpatient serum creatinine measurement between 7 days prior to admission and at least one inpatient creatinine data, with hospitalization greater than 24 h, as the AKI cohort. AKI was defined as an increase in serum creatinine by more than 0.3 mg/dl (26.5 µmol/l) within 48 h or an increase in serum creatinine to over 1.5 times that at baseline within the prior 7 days. The exclusion criteria of participants were: (1) patients who had end-stage renal disease; (2) patients who required renal replacement therapy (dialysis or renal transplantation); (3) change of serum creatinine didn’t attribute to AKI; (3) patients with hospital stay < 48 h or > 30 days; (4) patients with incomplete medical records or follow-up information; (5) Patients dead during 7 days; (6) patients with other comorbidities. Eventually, seventy-two individuals were selected for the final AKI cohort. At the same time, we collected information and samples of health volunteers during the same time period. The medical research ethics committee at the Yantai Affiliated Hospital of Binzhou Medical University approved this retrospective study. The information of participants was deidentified (Table 1). All participants or their legally authorized representatives have provided written informed consent. Recovery was defined as the serum creatinine concentrations falling below 90% of the baseline. Non-recovery was defined as plasma creatinine remaining above 90% of the serum creatinine at presentation or whenever the patient became dialysis dependent.

**Table 1:** Baseline data for the subjects

Group	control	AKI	*p*
Age (years, medium (25–75 percentile))	56.5 (53.0-60.8)	56.5 (53.0–61.0)	0.82
Gender (male, n, %)	37 (51.4)	43 (59.7)	0.31
BMI (mean ± standard deviation)	22.02 ± 2.10	22.33 ± 2.12	0.38
Moderate/severe AKI (n, %)	38 (52.8)	34 (47.2)	0.51

BMI, body mass index

The supernatants of peripheral venous blood samples were drawn at the time of AKI diagnosis. Briefly, fresh whole blood was collected in an anticoagulant tube, gently inverted several times to thoroughly mix with the anticoagulant, and placed in an upright position at 4℃ for 15 min. Then, after centrifugation at 3000 rpm for 10 min, the supernatant was taken and placed in a centrifuge tube. The obtained serum was quickly frozen in liquid nitrogen and transported to a -80℃. To perform RNA detection, samples were mobilized from the frozen condition.

### Cell culture

HK-2 proximal tubule epithelial cells were cultured at 37ºC, 5% CO_2_ in keratinocyte serum-free medium (Invitrogen, USA) supplemented with 10% FBS, 100 U/mL penicillin/streptomycin, 5 ng/mL epidermal growth factor and 50 µg/mL BPE. Cell experiments were performed at passages 5–12 when 70–80% confluent. HK-2 cells were seeded in 6-well plates in K-SFM without any supplements, and treated for 24 h with LPS (10 µg/mL). At the end of treatment, media was collected and filtered through 0.2 μm filter.

### Cell transfection

Knockdown of hsa_circ_0005519 in HK-2 was performed by transfecting the specific siRNAs (anti-CIRC) (Ribo Bio, China) at the concentration of 50 nM using X-treme siRNA transfection reagent (Roche Applied Science, Germany). Inhibition of miR-98-5p was performed by transfecting the cells with 50 nM miR-98-5p inhibitor (GenePharma, China), using the negative inhibitor served as controls (in-NC). The knockdown efficiency was assesse by reverse transcription-qPCR.

### RNA extraction and reverse transcription-qPCR

The extraction and quantification of total RNA were processed using TRIzol reagent (Thermo Fisher Scientific, USA) and a NanoDrop2000 spectrophotometer (Thermo Fisher Scientific, USA), respectively. Then, one µg of total RNA was converted into cDNA using the HiScript® II Q Select RT SuperMix for qPCR (Vazyme; China). Next, the qPCR reaction was performed using 1 µL cDNA in 5 µL SYBR®Green Master Mix (Vazyme; China), using GAPDH or U6 as an endogenous control. Primers used were listed in supplementary Table 1. The expression levels of RNA were calculated by the 2^−∆∆Ct^ method.

### In vitro inflammation-related factor assay

TNF-α, IL-6, and IL-10 were quantified in the filtrate of media from HK-2 cells 24 h after stimulation with LPS, by kit-based ELISA (R&D Systems, USA) according to standard protocols provided by the manufacturer.

### Assays for myeloperoxidase (MPO) activity and malondialdehyde (MDA) content

MPO activity and MDA content were analyzed to assess the oxidative stress level, using respective assay kits (Nanjing Jiancheng, China) following the manufacturer’s instructions.

### Cell viability assay

Cell viability was assayed by 3-(4,5-dimethylthiazol-2-yl)-2,5-diphenyltetrazolium bromide (MTT) assays. In brief, HK-2 cells were seeded into 96-well plates for 24-hour culture. MTT solution was then supplemented to the medium, followed by a culture of 2 h. Then, the medium was removed. Cells were resuspended and measured at 570 nm using a plate-reader (Bio-Rad Laboratories). The data was calculated as relative value of control group.

### Bioinformatics analysis

ENCORI was used for the prediction of downstream miRNAs for hsa_circ_0005519. The predicted miRNAs were then intersected with those differentially expressed miRNAs from GSE125305 dataset from GEO database (logFC>1 or logFC<-1, *P*.Value <0.05). ENCORI (clipExpNum >1), TargetScan (Total context + + score <-0.01), and miRDB (Target Score >60) were used for the prediction of miR-98-5p target genes. Then, the target genes were intersected with the AKI-related genes from GeneCards database (Relevance score more than the mean value). The common genes were subjected to OmicShare Tools for KEGG and GO enrichment analyses. The protein-protein interaction (PPI) network was constructed using STRING and Cytoscape. The hub genes were analyzed by Cytoscape.

### Dual-luciferase reporter assay

Dual-luciferase reporter plasmids, carrying wild-type or mutant hsa_circ_0005519 or IGF1R, were purchased from Hanbio (China). HK-2 cells were co-transfected with luciferase reporter plasmid and miR-93 mimics or mimic control using Lipofectamine 3000 (Invitrogen, USA). 48 h later, luciferase activity of cells was measured with a Dual Luciferase Reporter Gene Assay Kit (Beyotime, China) after 48 h. The ratio of FLUC activity to RLUC activity was calculated and then normalized to the ratio of the control group.

### Western blot analysis of IGF1R protein expression

For analysis of IGF1R protein expression, Western blotting technique was used. HK-2 cells were grown to 70% confluency. Next, the cells were washed with ice-cold PBS and lysed using radioimmunoprecipitation assay buffer (Absin, China) for 15 min at 4℃. The lysis solution was centrifuged at 12,000 rpm for 10 min. The supernatant was discarded, and precipitated protein was measured using the Pierce Coomassie (Bradford) Protein Assay Kit (Thermo Fisher Scientific, USA). Next, 20 µg of protein were loaded in each lane on a tris-glycine gel and transferred to nitrocellulose membranes. The menbrance were then blocked and incubated overnight with the indicated primary antibodies diluted 1:300 and 1:200 of the rabbit-derived polyclonal anti-IGF1R antibody (Santa Cruz, USA), and mouse β-actin (LI-COR Biosciences, USA). After secondary antibody incubation, membranes were scanned on an Odyssey infrared imaging system (LI-COR Biosciences, USA) at 800 nm wavelength. The scanned images were subjected to ImageJ for measurement of integrated density.

### Data analysis

The categorical parameters were compared between the control and AKI groups, using Pearson’s chi-squared test. The continuous variables were compared using the Mann-Whitney U test, after verification of non-normality by the Shapiro-Wilk test. The diagnostic capacity of hsa_circ_0005519 to differentiate patients with AKI from health, or recovered patients from those non-recovered ones, was evaluated using an receiver operating characteristic (ROC) curve-based analysis. The criterion for per-comparison significance was set at two-sided *p* < 0.05.

## Results

### Expression level of hsa_circ_0005519 in AKI

**Fig. 1:**
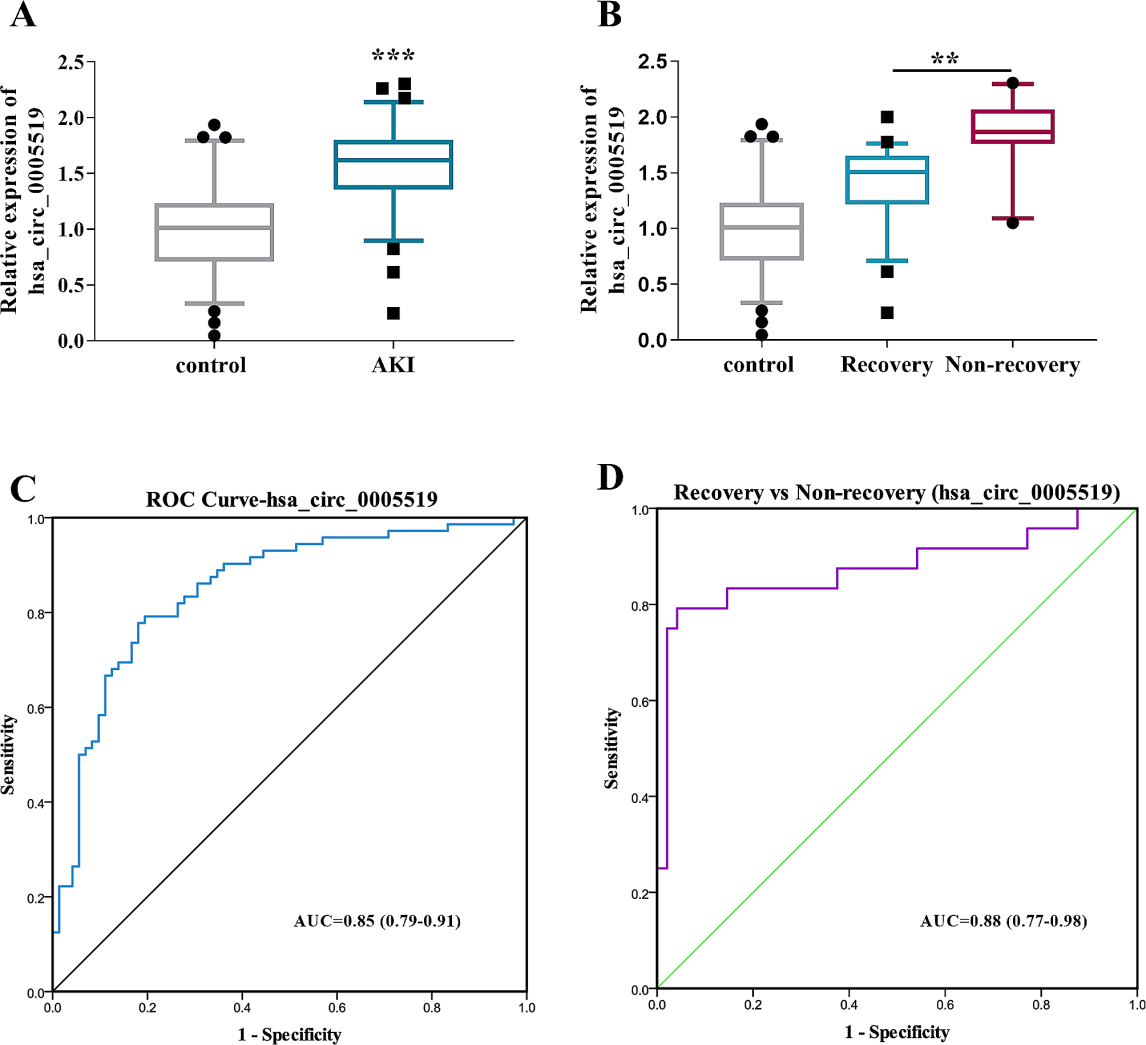
Reverse transcription-qPCR detection of hsa_circ_0005519 as a biomarker in AKI patient serum samples and control subjects. (**A**) Significant differences were observed between AKI patients (*n* = 72) and controls (*n* = 72) for hsa_circ_0005519. The figure reported the 5–95 percentile data of the normalized expression of hsa_circ_0005519. (**B**) Significant differences were observed between recovery (*n* = 48) and non-recovery (*n* = 24). The figure reported the 5–95 percentile data of the normalized expression of hsa_circ_0005519. (**C**) Receiver operating characteristic (ROC) curve comparing all AKI patients with controls for hsa_circ_0005519 gave an area under the curve (AUC) of 0.85. (**D**) ROC curve comparing recovery AKI patients with non-recovery AKI patients for hsa_circ_0005519 gave an AUC of 0.88. Statistical analysis comparing two groups was by Mann-Whitney U test. Data were presented as median and 5–95 percentile; **p < 0.01, ***p < 0.001. AKI, acute kidney injury

Our analysis cohort included 72 patients with AKI and 72 healthy controls (Table 1). Among the 72 AKI patients, Compared to patient serum in the control group, the serum in patients with AKI demonstrated a significant increase in hsa_circ_0005519 expression level (*p* < 0.001, Fig. 1A). In addition, significant differences were observed between recovered AKI patients and non-recovered AKI patients for serum hsa_circ_0005519 levels (*p* < 0.01, Fig. 1B), according to the 90-day follow-up information. RT-qPCR data for hsa_circ_0005519 were used to compare AKI patients to controls in the ROC curve analysis shown in Fig. 1C, giving an area under the curve (AUC) of 0.85. RT-qPCR data for hsa_circ_0005519 in AKI patients were used to compare AKI recovery and nonrecovery, with a ROC curve analysis giving an AUC of 0.88 (Fig. 1D).

### Hsa_circ_0005519 inhibited the viability in LPS-treated HK-2 cells

**Fig. 2:**
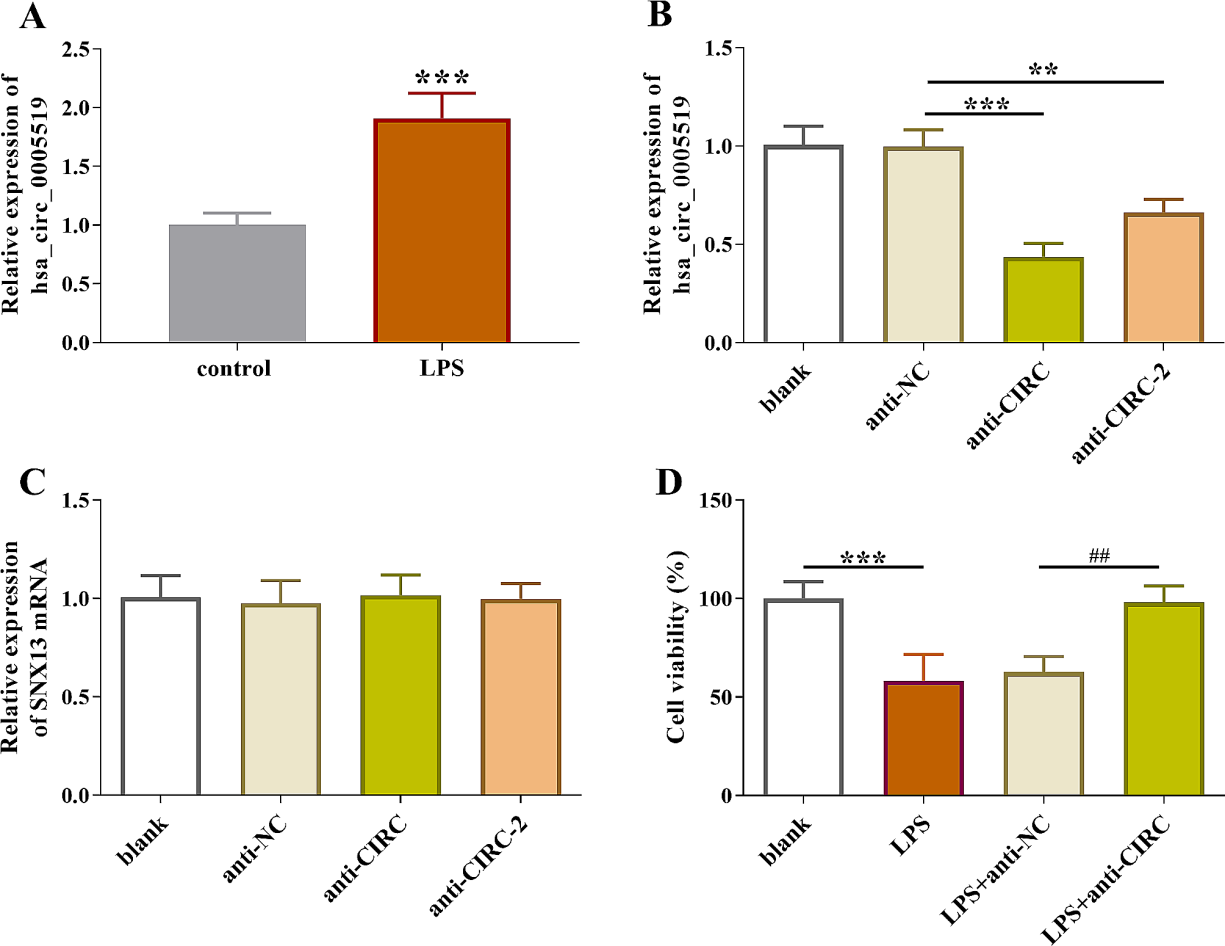
Elevated serum hsa_circ_0005519 was associated with cell viability. (**A**) Reverse transcription-qPCR analysis of expression of hsa_circ_0005519 in an in vitro model of AKI, LPS-induced HK-2 cells. ***p < 0.001. (**B**) Reverse transcription-qPCR analysis of hsa_circ_0005519 expression in transfected or non-transfected HK-2 cells. **p < 0.01, ***p < 0.001. (**C**) Reverse transcription-qPCR analysis of SNX13 (hsa_circ_0005519 host gene) expression in transfected or non-transfected HK-2 cells. (**D**) Inhibited hsa_circ_0005519 expression resulted in a decrease in HK-2 cell viability compared with siRNA negative control. Statistical analysis was completed using one-way ANOVA with post-hoc analysis of Turkey. ***p < 0.001, vs. blank. ##*p* < 0.01,vs. LPS + anti-NC

We then analyzed expression of hsa_circ_0005519 using LPS-driven cell injury model in vitro. Significant hsa_circ_0005519 (*p* < 0.001, Fig. 2A) was seen. To constraint the increase of hsa_circ_0005519 in AKI, siRNAs (anti-CIRC and anti-CIRC-2) were used to knockdown the expression level of hsa_circ_0005519 (*p* < 0.01, Fig. 2B). anti-CIRC was used for further research owing to its higher knockdown efficiency. The designed siRNA should specifically aim to hsa_circ_0005519, to avoid any interference with its linear host gene [16]. Notably, the siRNAs in this study showed no effects on expression of *SNX13* mRNA (hsa_circ_0005519 host gene) (Fig. 2C). MTT assay showed a decrease in cell viability after LPS-treatment, whereas knockdown of hsa_circ_0005519 restored the growth ability of HK-2 cells (*p* < 0.01, Fig. 2D).

### Effects of hsa_circ_0005519 on MPO, MDA, and inflammatory mediators in HK-2 cells

**Fig. 3:**
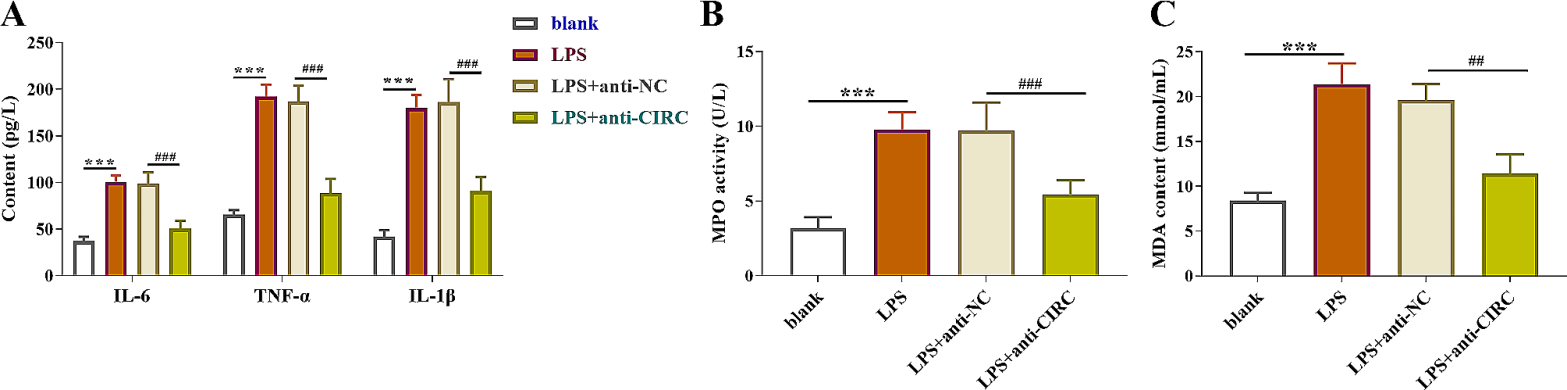
Hsa_circ_0005519 contributed to LPS-induced inflammatory response and oxidative stress in HK-2 cells. HK-2 cells were transfected with hsa_circ_0005519 siRNA (anti-CIRC), followed by culture in LPS. (**A**) Effect of hsa_circ_0005519 siRNA on the concentration of IL-6, TNF-α, and IL-1β was measured by ELISA. Effect of hsa_circ_0005519 siRNA on MPO activity (**B**) and MDA (**C**) content was detected using commercialized detection kits. Statistical analysis comparing two groups was by Mann-Whitney U test. ***p < 0.001, vs. blank. ##*p* < 0.01, ###*p* < 0.001,vs. LPS + anti-NC. LPS, lipopolysaccharide; MPO, myeloperoxidase; MDA, malondialdehyde; ELISA, enzyme-linked immunosorbent assay

ELISA tests was performed to detect the levels of pro-inflammatory mediators (IL-6, TNF-α, and IL-1β) involved in inflammation of the LPS treatment. The results gave a significant increase of these factors in the cells treated with LPS, compared to the blank group; but hsa_circ_0005519 inhibition significantly reduced the accumulation of their levels (*p* < 0.001, Fig. 3A). MDA serves as an indicator of lipid peroxidation, while MPO is an oxidative marker and an indicator of neutrophil infiltration into kidneys [17]. Cell damage caused by LPS was also illustrated by the increase in neutrophilic infiltration, presenting as the increased activity of MPO (Fig. 3B) and MDA (Fig. 3C), compared to the control group. Whereas knockdown of hsa_circ_0005519 was able to reduce the activity of MPO and MDA levels (*p* < 0.01, Fig. 3B and C).

### Prediction of the downstream miRNAs for hsa_circ_0005519

**Fig. 4:**
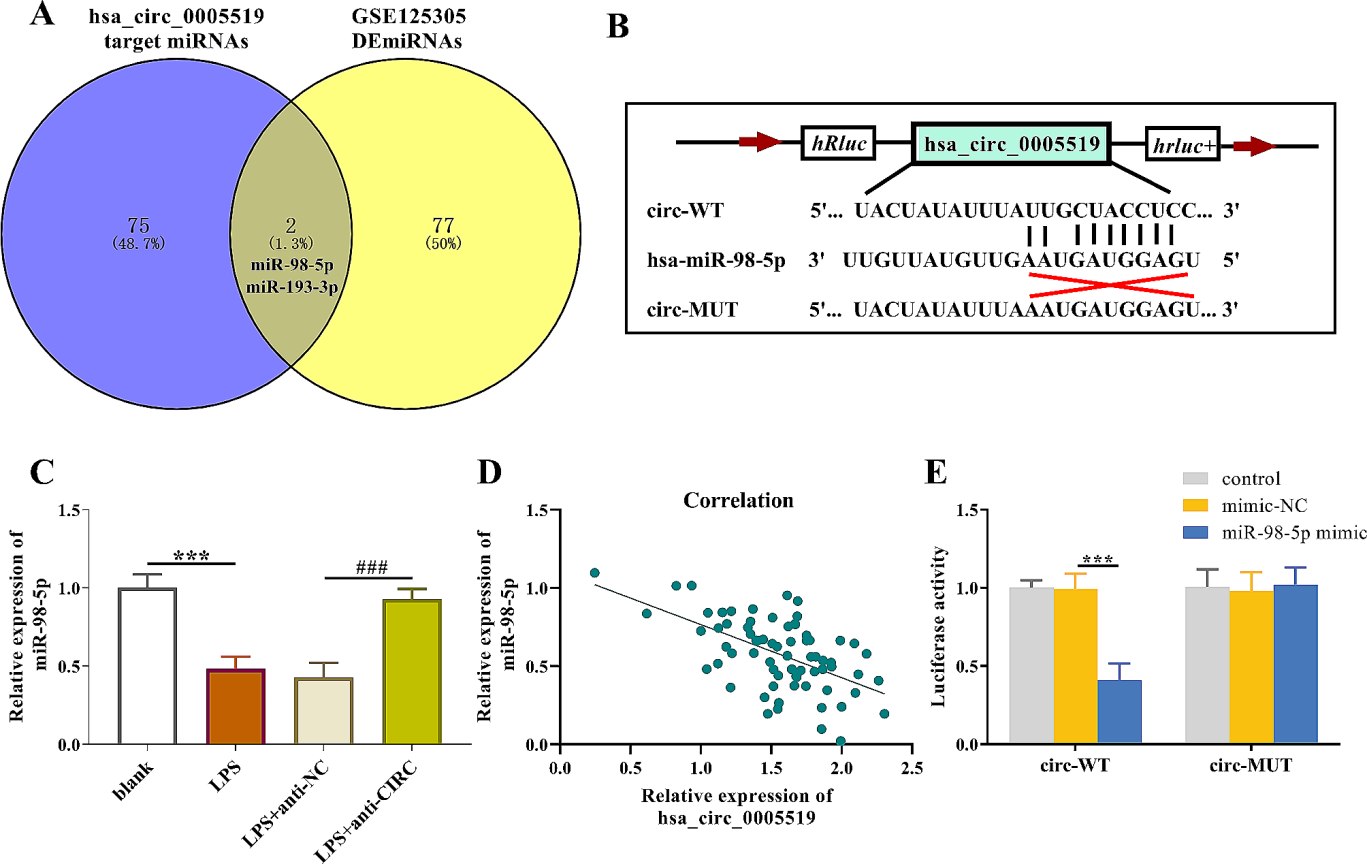
Hsa_circ_0005519 can sponge miR-98-5p. (**A**) The shared miRNAs between hsa_circ_0005519-targeted miRNAs and differentially expressed miRNAs (DEmiRNAs) in GSE125305. (**B**) The predicted binding sites of hsa_circ_0005519 in miR-98-5p. (**C**) Reverse transcription-qPCR analysis of miR-98-5p. (**D**) Correlation of hsa_circ_0005519 with miR-98-5p in AKI serum samples. (**E**) Luciferase reporter analysis of wild-type or mutant hsa_circ_0005519 under miR-98-5p overexpression

Then, prediction of the downstream target miRNAs was done by using ENCORI and GSE125305 dataset (Fig. 4A). The intersection showed 2 potential miRNAs, among which we found miR-98-5p, a kidney-protecting factor from oxidative stress and inflammation [18]. The binding sites between hsa_circ_0005519 and miR-98-5p were shown in Fig. 4B. RT-qPCR data for miR-98-5p showed that LPS can cause a reduction in miR-98-5p expression level while hsa_circ_0005519 siRNA can partly restore the miR-98-5p expression (Fig. 4C). Correlation analysis showed that hsa_circ_0005519 expression was negatively related to miR-93-5p expression (Fig. 4D). Luciferase reporter assays demonstrated that co-transfection of wild-type hsa_circ_0005519 luciferase reporter with miR-98-5p caused a sharp decrease in its luciferase activity, which wasn’t found for the mutant hsa_circ_0005519 luciferase reporter. These investigations indicated that miR-98-5p directly bound by hsa_circ_0005519 (Fig. 4E).

### Hsa_circ_0005519 promoted AKI by regulating miR-98-5p

**Fig. 5:**
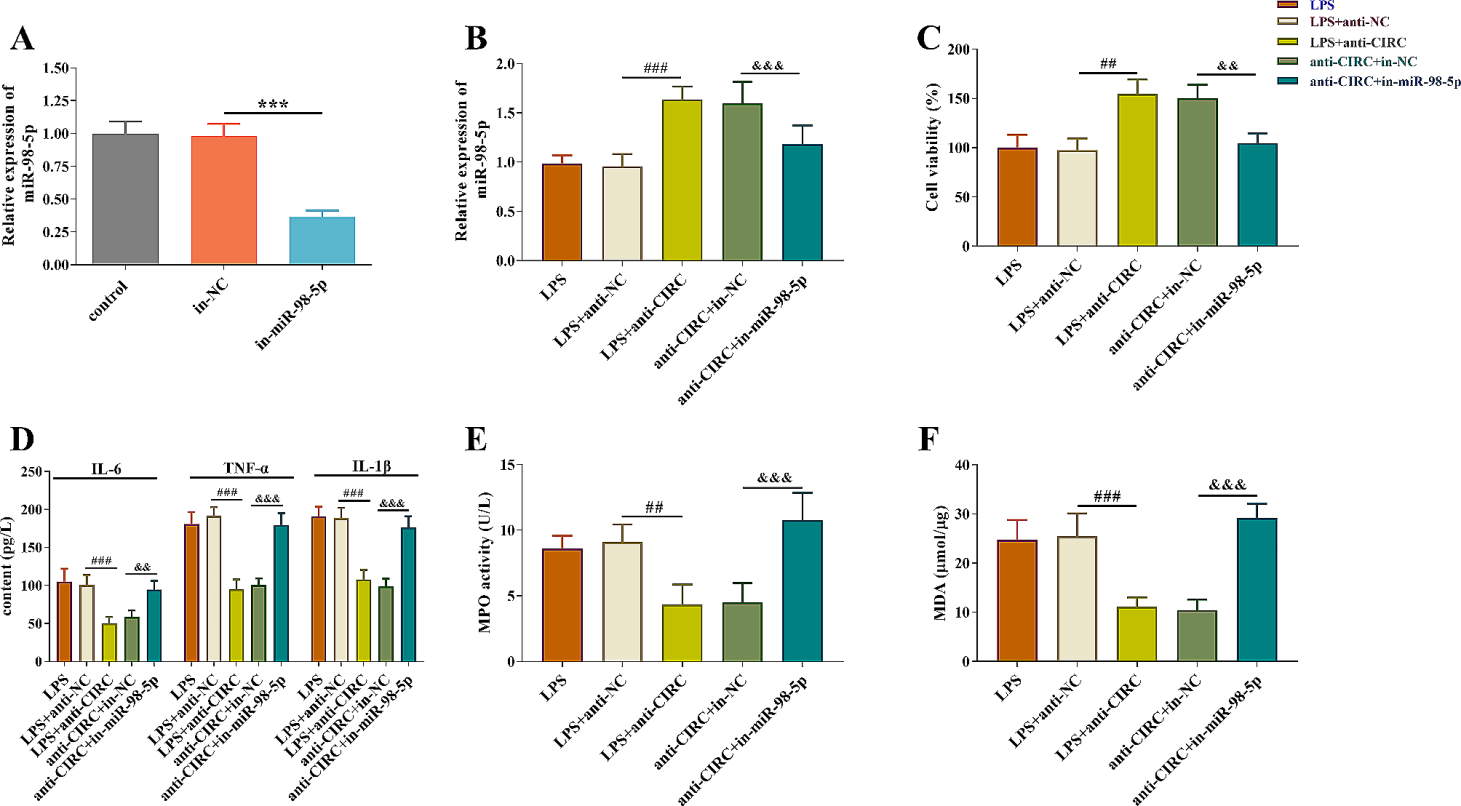
Effects of miR-98-5p inhibitor on cell viability, inflammatory response and oxidative stress regulated by hsa_circ_0005519 in LPS-treated HK-2 cells. (**A**) Reverse transcription-qPCR analysis of miR-98-5p after LPS stimulation. (**B**) Reverse transcription-qPCR analysis of miR-98-5p after transfection or co-transfection in LPS-treated HK-2 cells. (**C**) The changes in cell viability were determined using MTT assay. (**D**) The concentration of IL-6, TNF-α, and IL-1β were measured by ELISA. The MPO activity (**E**) and MDA (**F**) content were detected using commercialized detection kits. Statistical analysis comparing two groups was by Mann-Whitney U test. ***p < 0.001, vs. in-NC. ##*p* < 0.01, ###*p* < 0.001,vs. LPS + anti-NC. &&*p* < 0.01, &&&*p* < 0.001,vs. anti-CIRC + in-miR-98-5p. LPS, lipopolysaccharide; MPO, myeloperoxidase; MDA, malondialdehyde; ELISA, enzyme-linked immunosorbent assay

As miR-98-5p exhibited cell viability-protective effect in rat primary renal kidney cells [18], we wondered if miR-98-5p would alleviate the promotion effect of AKI. miR-98-5p inhibitor was introduced to decrease the expression level of miR-98-5p (*p* < 0.001, Fig. 5A). RT-qPCR data for miR-98-5p showed that the increased expression level caused by hsa_circ_0005519 inhibition was brought down by miR-98-5p inhibitor(*p* < 0.001, Fig. 5B). MiR-98-5p inhibition significantly reversed the effect of hsa_circ_0005519 knockdown on cell viability (*p* < 0.01, Fig. 5C). MiR-98-5p inhibitor can restore the proinflammatory cytokine expressions (*p* < 0.01, Fig. 5D), MPO activity (*p* < 0.01, Fig. 5E), and MDA content (*p* < 0.001, Fig. 5F) induced by hsa_circ_0005519 knockdown in HK-2 cells. These results indicated that hsa_circ_0005519 promoted LPS-induced changes, including cell viability, inflammatory response, and oxidative stress in HK-2 cells by sponging miR-98-5p.

### Prediction of the target genes for miR-98-5p

**Fig. 6:**
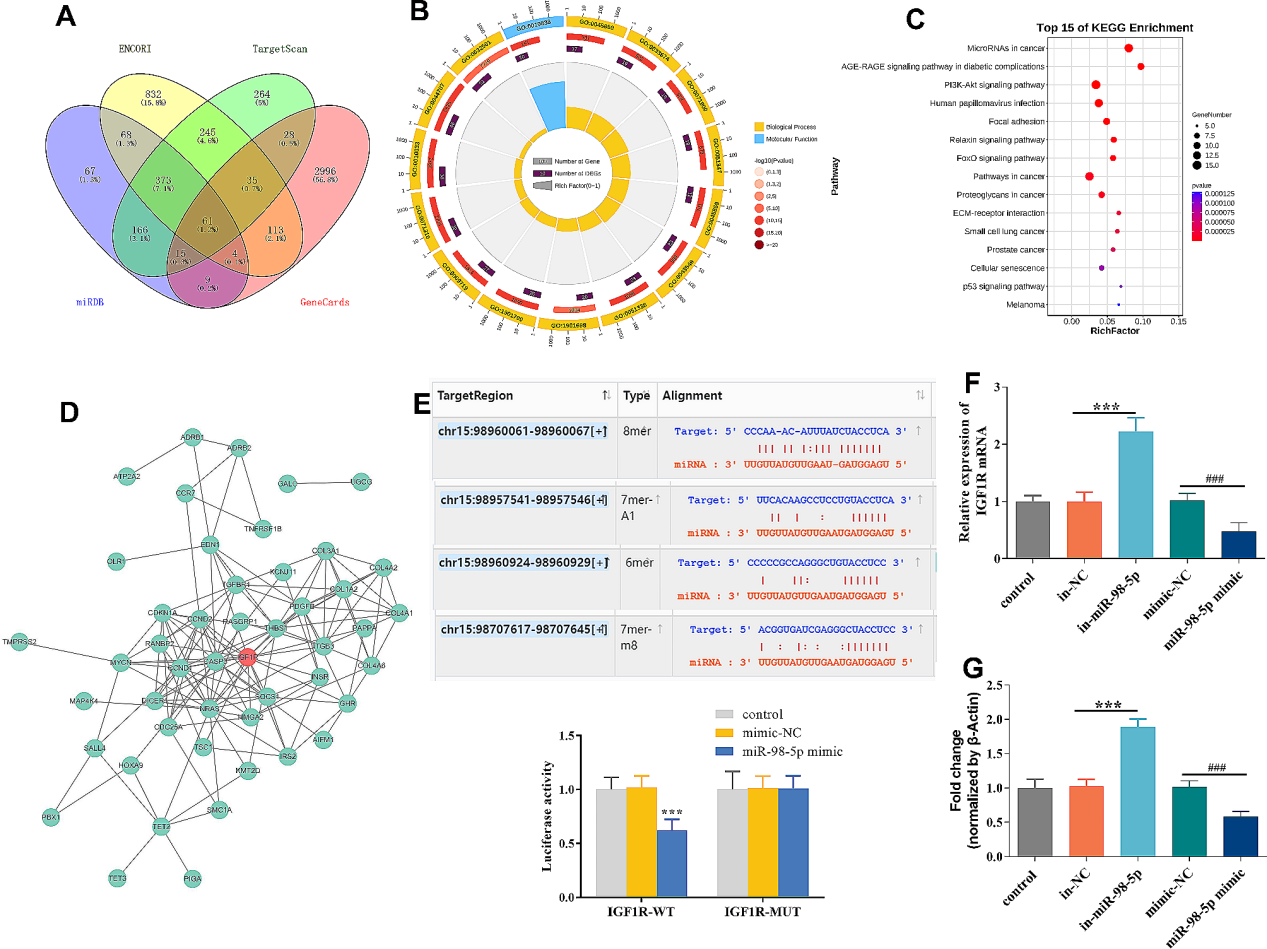
Prediction of miR-98-5p-targeting genes. (**A**) Screening of AKI-related genes targeted by miR-98-5p. (**B**) The Gene Ontology (GO) enrichment analysis of the 61 obtained genes. (**C**) The Kyoto Encyclopedia of Genes, and Genomes (KEGG) pathways enrichment analysis of the 61 obtained genes. (**D**) Protein-protein interaction for the 61 obtained genes. (**E**) The binding sites of miR-98-5p in IGF1R and luciferase reporter analysis. (**F**) Reverse transcription-qPCR for IGF1R mRNA expression. (**G**) Western blots reulats for IGF1R protein expression. Statistical analysis was completed using one-way ANOVA with post-hoc analysis of Turkey. ***p < 0.001. ###*p* < 0.001,vs. control

The target genes for miR-98-5p related to AKI were predicted by ENCORI, TargetScan, miRDB, and GeneCards (Fig. 6A). The GO enrichment was shown in Fig. 6B, including multicellular organismal process, developmental process, immune system process, response to stimulus, negative regulation of biological process, and growth. Key pathways enriched by these 61 genes were listed in Fig. 6C, including PI3K-Akt signaling pathway. Using Cytoscape software, IGF1R was identified as the hub gene among these 61 genes (Fig. 6D). Based on the four sets of binding sites between miR-98-5p and IGF1R, Luciferase reporter assays verified that miR-98-5p is directly targeted to IGF1R (Fig. 6E). MiR-98-5p overexpression reduced IGF1R mRNA and protein expression, while miR-98-5p knockdown increased IGF1R mRNA and protein expression (Fig. 6F and G).

## Discussion

Acute kidney injury is one of the debilitating syndromes, associated with an increased risk of mortality, cardiovascular events, and progression to chronic kidney disease [19]. New serum and urine biomarkers may allow earlier evidence and have exhibited possible roles for more accurate comparisons across populations [20]. Increasing evidence has shown that circRNAs play an important role in the progression of kidney diseases, including AKI [10]. It has been identified that a range of circRNAs are aberrantly expressed in AKI patients or animal models [21, 22]. However, the role of hsa_circ_0005519 in AKI is not clear. Previous results showed that the expression of hsa_circ_0005519 was upregulated in asphyxial newborns with AKI [15]. In this study, the expression level of hsa_circ_0005519 was also significantly upregulated in patients with AKI. The diagnostic value and function were further studied in AKI here. In the present study, hsa_circ_0005519 had a good performance in AKI diagnosis and recovery prediction. LPS treatment can inhibit the viability of HK-2 cells, whereas knockdown of hsa_circ_0005519 significantly decreased the viability changes of HK-2 cells induced by LPS. Moreover, the knockdown of hsa_circ_0005519 alleviated LPS-induced inflammation and alternations in MPO activity and MDA level.

Recent studies, clinical or experimental, have confirmed that the pathogenic stimulus of AKI converges cell death and cascading events [23]. In this current study, knockdown of hsa_circ_0005519 can alleviate the cell viability of HK-2 cells damaged by LPS, which may provide a new clue for AKI treatment. Tubular cell death during kidney injury can initiate an innate immune response, causing the secretion of proinflammatory cytokines and chemokines [24]. Additional cell deaths occurred because the responses would guide various inflammatory cells to the injury site, forming a cell death-inflammation cycle [25]. Accumulating evidence has shown that circRNAs participate in the inflammation process. It is reported that LPS can stimulate the severe inflammatory response and lead to an increase in pro-inflammatory factors, including TNF-a, IL-6, and IL-1B [26]. In this study, we found LPS stimulation increased the release of proinflammatory factors from HK-2 cells, whereas inhibition of hsa_circ_0005519 markedly suppressed LPS-caused inflammatory response. MDA is one of the final products of lipid peroxidation, which is commonly used as an oxidative marker [24]. Based on numerous studies, all injurious pathways for AKI converge on reactive oxygen species production and oxidative stress [27]. Oxidative damage is a common mechanism of AKI from any cause. Here, MPO is a pro-oxidant enzyme involved in oxidative stress during kidney injury. In this study, we found LPS can cause the accumulation of MPO, while hsa_circ_0005519 knockdown can attenuate LPS-induced MPO increase. Moreover, hsa_circ_0005519 inhibition markedly attenuated LPS-induced MDA (lipid peroxidation) increase. These observations indicated that hsa_circ_0005519 can promote inflammatory and oxidative stress during AKI.

Since the acknowledge of circRNA-associated competitive endogenous RNA (ceRNA) regulatory networks, plenty of studies have demonstrated that circRNA molecules that compete for shared target RNAs are important components of gene regulation in many cell-related processes [28]. Different circRNAs can act as ceRNA and modulate the expression of mRNAs via sponging miRNAs. Deciphering the molecular mechanisms associated with ceRNA in AKI allows us to obtain insights into the potential of circRNAs as biomarkers for this disease. Here, we predicted and verified that hsa_circ_0005519 can sponge miR-98-5p. MiR-98-5p can reverse the viability-inhibiting and inflammation/oxidative stress-induced effects of hsa_circ_0005519 in HK-2 cells. Then, IGF1R was screened as the hub gene of miR-98-5p in AKI. MiR-98-5p may be potentially employed as a biomarker for human kidney diseases [29]. MiR-98-5p-overexpressed microvesicles secreted by endothelial progenitor cells can inhibit oxidative stress and inflammation in rat primary renal kidney cell, via moderating IGF1R [18]. IGF-I administration has been reported to increase macrophage infiltration and inflammatory response in rats with ischemic acute renal failure, increasing the mortality rate of rats [30]. PI3K/Akt pathway was one of the enriched pathways by miR-98-5p-targeting genes in this study. This was in line with the previous study, which showed miR-98-5p can activate the PI3K/Akt/eNOS pathway by regulating IGF1R [18]. Therefore, hsa_circ_0005519 knockdown may protect the renal cells from oxidative stress, inflammation and inhibition of cell viability via sponging miR-98-5p and then moderate IGF1R. However, we just preliminarily demonstrated that IGF1R was a miR-98-5p target gene. More data will be needed to further prove that IGF1R is involved in the role of hsa_circ_0005519 in AKI.

## Conclusions

In conclusion, our findings suggested a contributing role of hsa_circ_0005519 in LPS-induced AKI by sponging miR-98-5p and then moderating IGF1R. Hsa_circ_0005519 exhibited a possible role in distinguishing patients with AKI across populations. Therefore, hsa_circ_0005519 may be potentially employed as a biomarker and therapy target for AKI.

### Electronic supplementary material

Below is the link to the electronic supplementary material.

Supplementary Material 1

## Data Availability

The datasets used and/or analysed during the current study are available from the corresponding author on reasonable request.
