# Supplementary material for: Circular RNA hsa_circ_0005519 contributes to acute kidney injury via sponging microRNA-98-5p
Source: BMC Nephrol. 2024 Mar 19;25:107. doi: 10.1186/s12882-024-03544-8 (PMC10949765; doi:10.1186/s12882-024-03544-8)
Supplement: Supplementary file 1 — Supplementary Material 1 [file 12882_2024_3544_MOESM1_ESM.docx]

Supplementary Table 1 Primer sets used in this study

| Primer set | Forward Primer | Reverse Primer |
| --- | --- | --- |
| hsa_circ_0005519 | TTGTCAGGGAAATCCTTGCA | CCAGTCTATTTCTTTTGACCATCCA |
| SNX13 mRNA | AGAGAAAACTGCCCCCATCT | CACAGCCCTTAAAGCACACA |
| IGF1R mRNA | TGTGTGGACCGTGACTTCTG | GGACCTTCACAAGGGATGCA |
| miR-98-5p | TGCTTGAGGTAGTAAGTTG | ATCCAGTGCGTGTCGTG |
| U6 | CTCGCTTCGGCAGCACATATA | ACGCTTCACGAATTTGAGTGTC |
| GAPDH | GAACGGGAAGCTCACTGG | GCCTGCTTCACCACCTTCT |
